# Psychosocial working conditions, asthma self-management at work and asthma morbidity: a cross-sectional study

**DOI:** 10.1186/s13601-019-0264-9

**Published:** 2019-05-09

**Authors:** Katherina Heinrichs, Stefan Hummel, Jalal Gholami, Konrad Schultz, Jian Li, Aziz Sheikh, Adrian Loerbroks

**Affiliations:** 1 Institute of Occupational, Social, and Environmental Medicine, Centre for Health and Society, Faculty of Medicine, University of Düsseldorf, Universitätsstraße 1, 40225 Düsseldorf, Germany; 2 MEDIAN Klinik Heiligendamm, Kinderstrand 1, 18209 Bad Doberan, Germany; 3 Nordseeklinik Borkum der DRV Rheinland, Bubertstraße 4, 26757 Borkum, Germany; 4 Klinik Bad Reichenhall der DRV Bayern Süd, Salzburger Str. 8, 83435 Bad Reichenhall, Germany; 5 Department of Environmental Health Sciences, Fielding School of Public Health, Los Angeles, CA 90095-1772 USA; 6 School of Nursing, University of California Los Angeles, Los Angeles, CA 90095-1772 USA; 7 Usher Institute of Population Health Sciences and Informatics, University of Edinburgh, Old Medical School, Teviot Place, Edinburgh, EH8 9AG UK

**Keywords:** Asthma, Quality of life, Psychosocial working conditions, Occupational stress, Self-management, Workplace

## Abstract

**Background:**

Adverse psychosocial working conditions—in particular poor job decision latitude and poor social support at work—may impair the effective implementation of asthma self-management behaviour at work and may be associated with increased asthma morbidity. In this study, we investigate for the first time the association of job decision latitude and social support at work with (1) four asthma-specific self-management behaviours at work (i.e., physical activity, trigger avoidance, acute symptom management, and communication) and with (2) asthma morbidity.

**Methods:**

A total of 221 employees with asthma recruited through three rehabilitation clinics completed questionnaires (response rate = 29.3%). Job decision latitude and social support were measured using items from the Copenhagen Psychosocial Questionnaire. The four asthma self-management behaviours were mainly assessed by self-developed items. We used the Asthma Control Test and the Marks Asthma Quality of Life Questionnaire to measure asthma morbidity. We dichotomized all variables and conducted logistic regression analyses to calculate odds ratios with 95% CIs.

**Results:**

Low job decision latitude and low social support were significantly associated with poorer trigger avoidance (odds ratios ≥ 2.09) and poorer acute symptom management (odds ratios ≥ 2.29); low social support was further related to significantly less communication (odds ratio = 2.82). Low job decision latitude and low social support were also associated with significantly poorer asthma control (odds ratios ≥ 1.95) and poorer asthma-specific quality of life (odds ratios ≥ 2.05). The relationships with asthma morbidity were attenuated after adjustment for the four asthma self-management behaviours (odds ratios ranging from 1.1 to 1.9).

**Conclusions:**

Adverse psychosocial working conditions are associated with poorer asthma self-management behaviour at work and with increased asthma morbidity. The latter association may be mediated by asthma self-management behaviour.

*Trial registration* German Clinical Trials Register, registration number: DRK S00011309, date of registration: 22.12.2016.

**Electronic supplementary material:**

The online version of this article (10.1186/s13601-019-0264-9) contains supplementary material, which is available to authorized users.

## Introduction

Effective asthma self-management behaviour (SMB)—e.g., symptom prevention or acute symptom management [1]—can improve the control of asthma and its prognosis [2]. The actual implementation of SMB is partially determined by contextual factors [1], e.g., by the working conditions employees with asthma are exposed to [3].

A qualitative interview study among employees with asthma suggested that two specific elements of well-established work stress models play a crucial role in the effective implementation of asthma SMB at work [3]: job decision latitude (JDL; i.e., the control over one’s tasks and when and how to complete them [4, 5]) and social support by colleagues and line-managers [6–8]. The two factors subjectively related to different domains of asthma SMB: high JDL seemed to promote symptom prevention, symptom monitoring, and acute symptom management, whereas support was perceived to facilitate symptom prevention, acute symptom management and communication (e.g., self-disclosure of one’s condition) [3].

Low levels of JDL and social support are considered important contributors to work stress [4, 5], which has been related to an increased incidence and prevalence of asthma [9–12] and which may also be associated with increased asthma morbidity [13, 14]. The latter association may be explained by direct physiological stress responses (e.g., decreased corticosteroid sensitivity due to long-term stress exposure [15]) and/or by behavioural pathways (i.e., poorer asthma self-management).

In this epidemiological study, we sought to build on the findings from our prior qualitative study and investigate the associations of JDL and social support with different types of asthma SMB [3]. Furthermore, we aimed to also test the relationships between the two psychosocial working conditions and asthma morbidity (i.e., asthma control and asthma-specific quality of life) and to investigate possible mediation of these associations by asthma SMB.

## Methods

### Study population

We recruited inpatients with asthma from three pulmonary rehabilitation clinics (i.e., Median Klinik Heiligendamm, Nordseeklinik Borkum der DRV Rheinland, and Klinik Bad Reichenhall der DRV Bayern Süd). In Germany, pulmonary rehabilitation aims to stabilize patients with long-term conditions to ensure social participation in private and professional life, including workability. This treatment is mostly financed by the health insurance carrier or the German Pension Fund.

Senior physicians screened the records of patients who were newly admitted or about to be admitted for an established asthma diagnosis while excluding patients with comorbid chronic obstructive pulmonary disease (COPD) to strengthen our case definition [16]. The inclusion and exclusion of participants were solely based on the admission diagnoses as stated in the patients’ files. There is evidence that patients with asthma and COPD behave differently concerning their disease self-management at work. In a qualitative study by Boot et al. [17] who investigated different coping profiles among employees with asthma or COPD, some of the workers with COPD were reported to enter a so-called “worried adaptation profile”, which was characterized by preoccupations with their health, adaptation to the experienced limitations and slower working. By contrast, employees with asthma did not display that adaptation profile, but formed, amongst others, a separate group of “eager workers”, who were reported to be motivated, leading a healthy life, adjusting their medication when necessary and reluctant to take sick leave [17]. Further, the authors postulated that patients with progressive diseases like COPD are more likely to suffer from adverse working conditions [17]. If we had included both illnesses in our study, we would have mixed up two different processes concerning self-management at work and would have reduced the generalizability of our results to either condition. Questionnaires with stamp-addressed envelopes were sent out to the patients as soon as they were registered (Bad Reichenhall) or handed to patients personally by the senior physician in their first week of inpatient treatment (Heiligendamm and Borkum). Out of 755 questionnaires, 221 eligible questionnaires were returned between October 2017 and May 2018 (response rate = 29.3%).

Patients’ questionnaires were eligible for analyses when the following criteria were met:

1. a diagnosis of and current treatment for asthma, but not COPD, according to admission diagnoses and confirmed by patient reports;
2. employment of at least 20 working hours per week in the last 6 months;
3. having worked with the diagnosis for at least 6 months.

Criteria 2 and 3 were applied to ensure that the participants had worked for a reasonable period of time with asthma.

### Ethical considerations

The participants provided written informed consent before taking part in the study. Our study received ethical approval by the Institutional Review Board of the Medical Faculty of the Heinrich-Heine University of Düsseldorf (no. 5764R).

### Measures

#### Psychosocial working conditions

We assessed the working conditions that were considered most important by the participants of our prior interview study [3]—namely, JDL and social support at work. Both constructs are elements of the well-established job demand-control-support model [4, 5] and were measured by the German version of the Copenhagen Psychosocial Questionnaire [18]. We combined the scales “Influence at work” (3 items) and “Degree of freedom at work” (2 items) into a sum-score to operationalize JDL (Cronbach’s alpha: α = .78). The four-item scale “social support” (Cronbach’s alpha: α = .90) was used to measure support. Sum-scores of each variable were dichotomized at the bottom tertile to define adverse working conditions. For methodological details, please see Additional file 1.

We sought to assess additional asthma-specific working conditions that emerged from our prior study [3]. As an appropriate instrument was lacking, we constructed a novel scale: the “Determinants of work-related asthma self-management (DReAM)” scale. The items were devised based on our qualitative findings and comprised statements concerning asthma-specific working conditions (e.g., “In case of acute asthma symptoms, I promptly take breaks in my everyday work”). They were optimized by cognitive interviews and further reduced in psychometric analyses (see Additional file 1 for details). The final scale comprised seven items all of which loaded on a single factor (Cronbach’s alpha = .83). For analyses, the DReAM-items (shown in Table 1) were combined into a sum-score which was dichotomized at the top tertile of the score distribution to define adverse working conditions.

**Table 1:** “Determinants of work-related asthma self-management (DReAM)” items (n = 221)

Item	Participants reporting adverse working conditions	
	n	%^a^
In my everyday work, I am able to avoid allergens or other triggers that are relevant to me	163	74.1
Since my condition is invisible to others, I do not receive any support in managing my asthma in my everyday work	148	67.6
In my everyday work, I can take a break anytime if necessary for my asthma management	136	62.1
In my everyday work, I can go home early or stay home for one day without a sick note whenever I need to manage my asthma	127	58.3
In my everyday work, I can manage my asthma the way I need to because I can withdraw from others for my asthma management	125	57.3
In my everyday work, I am NOT able to manage my asthma as I need to, because there is nobody to take over my tasks	106	48.2
I feel accepted by my line-manager concerning my asthma and the related limitations	62	28.8

^a^of eligible cases

#### Asthma self-management

Based on a model of four domains of asthma SMB [1], we aimed to measure symptom prevention, acute symptom management, and communication. The fourth domain (i.e., symptom monitoring) did not seem to be related to work (e.g., peak flow metre use) [3]. The domain symptom prevention was measured by items on trigger avoidance and physical activity. Items assessing acute symptom management covered, for instance, reliever medication use, breathing techniques, or taking breaks. Communication mainly referred to self-disclosure.

We used an item of the Survey of Health, Ageing and Retirement in Europe to assess physical activity [19, 20]: “How often do you engage in vigorous physical activity, such as sports, heavy housework, or a job that involves physical labour?”. We defined adequate physical activity as “more than once a week” (vs. once a week, one to three times a month, hardly ever, or never) which represents a level of physical activity that is beneficial to asthma morbidity [19, 20].

To assess further domains of asthma SMBs [1], we devised items based on our prior qualitative work [3]. We used a response format which had been applied before and had been assessed as useful by asthma patients [21]: “Yes, I do this”, “No, but I would like to”, and “No, I do not need this”. Based on the results of cognitive interviews, we reduced the item pool to its final set of ten items covering trigger avoidance (1 item), acute symptom management (7 items), and communication (2 items). To dichotomize the answers, we defined “No, but I would like to” as an unfulfilled need (scored as 1) and “Yes, I do this” or “No, I do not need this” as a fit of need and working conditions (scored as 0). Also the response option “Item does not apply to me” was scored as 0. For more information on the subscales and the strategy of dichotomization, please see Additional file 1.

#### Asthma morbidity

To assess asthma control, we used the Asthma Control Test [22]. The resulting sum-score was categorized into uncontrolled (< 20 points) versus controlled (≥ 20 points) [21, 23]. We measured asthma-specific quality of life using the Marks Asthma Quality of Life Questionnaire [24]. The total score was dichotomized at its top tertile to indicate impaired quality of life [21]. For methodological details, see Additional file 1.

### Data analysis

We used SPSS 25 to conduct logistic regression analyses. The reference categories were high JDL, high support and good working conditions according to the DReAM-scale, respectively. The dependent variables were reported physical activity once a week or less, unfulfilled needs regarding trigger avoidance, acute symptom management, and communication, poor asthma control, and impaired asthma-specific quality of life according to the different dichotomization strategies reported in Additional file 1. We initially estimated unadjusted odds ratios (ORs) and 95% confidence intervals (CIs), which were subsequently adjusted for age, sex, highest educational degree, body mass index, and smoking status (never, current, former) (i.e., labelled “Model A”). To investigate potential mediating effects of different asthma SMBs on the relationships of JDL or support with asthma control or asthma-specific quality of life, we added the respective asthma SMBs to the adjusted model (i.e., Model A) and re-ran the analyses.

## Results

### Sample description

Table 2 shows the characteristics of our sample (n = 221). The mean age was 50.6 years (standard deviation = 8.7 years). The gender distribution was fairly balanced (53.8% women). Over 60% of the sample had a secondary school degree or higher. More than one-third of the sample was obese. Almost two-thirds of the sample reported uncontrolled asthma.

**Table 2:** Sample characteristics (n = 221)

Characteristic	
Age (years)	
Mean (SD)	50.6 (8.7)
Missing values; n (%)	2 (.9)
Sex	
Women; n (%)	119 (53.8)
Men; n (%)	102 (46.2)
Missing values; n (%)	–
Highest school degree	
Lower than secondary school degree; n (%)	82 (37.1)
Secondary school degree; n (%)	74 (33.5)
Higher than secondary school degree; n (%)	62 (28.1)
Missing values; n (%)	3 (1.4)
Body mass index (BMI)	
Underweight, normal (BMI < 25); n (%)	61 (27.6)
Overweight (25 ≤ BMI < 30); n (%)	81 (36.7)
Obese (BMI ≥ 30); n (%)	76 (34.4)
Missing values; n (%)	3 (1.4)
Smoker	
No, never; n (%)	110 (49.8)
No, not anymore; n (%)	96 (43.4)
Current; n (%)	13 (5.9)
Missing values; n (%)	2 (.9)
Job Decision Latitude (JDL)^a^	
Mean (SD)	45.61 (22.83)
Missing values; n (%)	2 (.9)
Support^a^	
Mean (SD)	53.65 (25.65)
Missing values; n (%)	12 (5.4)
DReAM (working conditions)^b^	
Mean (SD)	18.50 (5.05)
Missing values; n (%)	1 (.5)
Physical activity	
More than once a week; n (%)	144 (65.2)
Once a week or less; n (%)	75 (33.9)
Missing values; n (%)	2 (.9)
Trigger avoidance^c^	
Mean (SD)	.6 (.5)
Missing values; n (%)	1 (.5)
Acute asthma symptom management^d^	
Mean (SD)	2.3 (2.0)
Missing values; n (%)	5 (2.3)
Communication^e^	
Mean (SD)	.3 (.6)
Missing values; n (%)	4 (1.8)
Asthma control^f^	
Controlled	76 (34.4)
Uncontrolled	145 (65.6)
Missing values	–
Asthma-specific quality of life^g^	
Mean (SD)	4.4 (1.8)
Missing values; n (%)	1 (.5)

*SD* standard deviation

^a^“Measured by the Copenhagen Psychosocial Questionnaire [37], range: 0–100, higher values indicating higher levels of JDL and support

^b^“Determinants of work-related asthma self-management (DReAM)” measured by 7 self-constructed items, range: 7–28, lower values indicating better working conditions

^c^Measured by 1 self-constructed item, range: 0 = fit of need and conditions versus 1 = need unfulfilled

^d^Measured by 7 self-constructed items, range: 0 = fit of need and conditions to 7 = all needs unfulfilled

^e^Measured by 2 self-constructed items, range: 0 = fit of need and conditions to 2 = both needs unfulfilled

^f^Measured by the Asthma Control Test [22], range: 5–25, higher values indicating higher levels of asthma control

^g^Measured by the Marks Asthma Quality of Life Questionnaire [24], range: 0–10, lower values indicating better quality of life

### Psychosocial working conditions and asthma SMBS at work

Table 3 shows estimates of the relationships between working conditions and asthma SMBs. In unadjusted analyses, employees with asthma who experienced low levels of JDL did not report less physical activity than employees with asthma who experienced high levels of JDL (OR = 1.57; 95% CI 0.87–2.84), but they reported more unfulfilled needs concerning trigger avoidance (OR = 2.30; 95% CI 1.23–4.31) and acute symptom management (OR = 5.83; 95% CI 2.76–12.31). There was no evidence of relationship between JDL and communication (OR = 1.57; 95% CI 0.77–3.18). Analyses adjusted for confounders (Model A) yielded comparable results.

**Table 3:** ORs and 95% CIs for different domains of asthma SMBs according to JDL and support (n = 221)

	Asthma symptom prevention								Acute asthma symptom management (unfulfilled needs)				Communication (unfulfilled needs)			
	Physical activity (once a week or less)				Trigger avoidance (unfulfilled need)				Unadjusted		Adjusted Model A^a^		Unadjusted		Adjusted Model A^a^	
	Unadjusted		Adjusted Model A^a^		Unadjusted		Adjusted Model A^a^									
	OR	95% CI	OR	95% CI	OR	95% CI	OR	95% CI	OR	95% CI	OR	95% CI	OR	95% CI	OR	95% CI
JDL																
High	Ref.	–	Ref.	–	Ref.	–	Ref.	–	Ref.	–	Ref.	–	Ref.	–	Ref.	–
Low	1.57	.87, 2.84	1.60	.84, 3.03	2.30	1.23, 4.31	2.05	1.07, 3.94	5.83	2.76, 12.31	6.32	2.83, 14.13	1.57	.77, 3.18	1.63	.79, 3.39
Support																
High	Ref.	–	Ref.	–	Ref.	–	Ref.	–	Ref.	–	Ref.	–	Ref.	–	Ref.	–
Low	1.17	.64, 2.13	1.42	.74, 2.73	2.09	1.13, 3.84	2.25	1.18, 4.31	2.29	1.08, 4.83	1.87	.85, 4.15	2.82	1.37, 5.84	2.95	1.38, 6.30
DReAM																
Good	Ref.	–	Ref.	–	Ref.	–	Ref.	–	Ref.	–	Ref.	–	Ref.	–	Ref.	–
Adverse	1.91	1.05, 3.46	2.68	1.37, 5.23	5.25	2.56, 10.79	6.54	2.98, 14.37	5.55	2.64, 11.69	4.67	2.14, 10.21	2.79	1.38, 5.63	2.87	1.37, 6.02

*OR* odd ratio, *CI* confidence interval, *SMB* self-management behaviour, *JDL* job decision latitude, *DReAM* determinants of work-related asthma self-management

^a^Model A: adjusted for age, sex, highest educational degree, body mass index, and smoker status

Low levels of social support (vs. high support) were associated with more unfulfilled needs concerning trigger avoidance (OR = 2.09; 95% CI 1.13–3.84), acute symptom management (OR = 2.29; 95% CI 1.08–4.83), and communication (OR = 2.82; 95% CI 1.37–5.84). There was no evidence of a relationship between support and physical activity (OR = 1.17; 95% CI 0.64–2.13). One association was attenuated in adjusted analyses, which is between support and acute symptom management (adjusted OR = 1.87; 95% CI 0.85–4.15).

Employees with asthma who reported adverse psychosocial working conditions according to the DReAM scale reported less physical activity (OR = 1.91; 95% CI 1.05–3.46) and more unfulfilled needs concerning trigger avoidance (OR = 5.25; 95% CI 2.56–10.79), acute symptom management (OR = 5.55; 95% CI 2.64–11.69), and communication (OR = 2.79; 95% CI 1.38–5.63). Adjusted analyses delivered similar results.

### Psychosocial working conditions and asthma morbidity

Results regarding the relationships between working conditions and asthma control are shown in Table 4. In unadjusted analyses, employees with asthma who experienced poor psychosocial working conditions in terms of JDL, support, and DReAM were more likely to report poor asthma control (OR = 2.14; 95% CI 1.12–4.10, OR = 1.95; 95% CI 1.04–3.68, and OR = 1.90; 95% CI 1.01–3.60, respectively).

**Table 4:** ORs and 95% CIs for asthma control according to JDL and support (n = 221)

	Poor asthma control													
	Unadjusted model		Adjusted Model A^a^		Adjusted Model B^b^		Adjusted Model A and physical activity		Adjusted Model A and trigger avoidance		Adjusted Model A and acute asthma symptom management		Adjusted Model A and communication	
	OR	95% CI	OR	95% CI	OR	95% CI	OR	95% CI	OR	95% CI	OR	95% CI	OR	95% CI
JDL														
High	Ref.	–	Ref.	–	Ref.	–	Ref.	–	Ref.	–	Ref.	–	Ref.	–
Low	2.14	1.12,4.10	2.21	1.12, 4.35	1.61	.75, 3.44	2.21	1.12, 4.37	1.96	.98, 3.89	1.68	.79, 3.57	2.28	1.13, 4.59
					− 27.15% change vs. adjusted Model A		0% change vs. adjusted Model A		− 11.31% change vs. adjusted Model A		− 23.89% change vs. adjusted Model A		+ 3.17% change vs. adjusted Model A	
Support														
High	Ref.	–	Ref.	–	Ref.	–	Ref.	–	Ref.	–	Ref.	–	Ref.	–
Low	1.95	1.04, 3.68	1.94	1.00, 3.76	1.52	.73, 3.18	1.94	.999, 3.76	1.73	.88, 3.39	1.74	.87, 3.49	1.85	.93, 3.68
					− 21.65% change vs. adjusted Model A		0% change vs. adjusted Model A		− 10.82% change vs. adjusted Model A		− 10.31% change vs. adjusted Model A		− 4.64% change vs. adjusted Model A	
DReAM														
Good	Ref.	–	Ref.	–	Ref.	–	Ref.	–	Ref.	–	Ref.	–	Ref.	–
Adverse	1.90	1.01, 3.60	1.86	.95, 3.66	1.16	.53, 2.55	1.89	.95, 3.75	1.49	.72, 3.06	1.34	.65, 2.78	1.79	.89, 3.59
					− 37.63% change vs. adjusted Model A		+ 1.61% change vs. adjusted Model A		− 19.89% change vs. adjusted Model A		− 27.96% change vs. adjusted Model A		− 3.76% change vs. adjusted Model A	

*OR* odd ratio, *CI* confidence interval, *JDL* job decision latitude, *DReAM* determinants of work-related asthma self-management

^a^Model A: adjusted for age, sex, highest educational degree, body mass index, and smoker status

^b^Model B: adjusted for age, sex, highest educational degree, body mass index, smoker status, physical activity, trigger avoidance, acute asthma symptom management, and communication

Analyses adjusted according to Model A yielded comparable results. After additional adjustment for all SMBs (Model B), all associations were attenuated as compared to Model A (ORs dropped by 27.2%, 21.7%, and 37.6%, respectively). Adjustment for single SMBs showed that this attenuation was most pronounced after adjustment for acute symptom management (− 23.9%, − 10.3%, and − 28.0%, respectively) and for trigger avoidance (− 11.3%, − 10.8%, and − 19.9%, respectively).

Table 5 displays the results regarding the relationships between working conditions and asthma-specific quality of life. In unadjusted analyses, employees with asthma who experienced poor psychosocial working conditions in terms of JDL, support, and DReAM reported impaired asthma-specific quality of life (OR = 2.05; 95% CI 1.13–3.72, OR = 2.56; 95% CI 1.40–4.67, and OR = 2.33; 95% CI 1.28–4.23, respectively) compared to employees with asthma who experienced good working conditions.

**Table 5:** ORs and 95% CIs for asthma-specific quality of life according to JDL and support (n = 221)

	Low asthma-specific quality of life													
	Unadjusted model		Adjusted Model A^a^		Adjusted Model B^b^		Adjusted Model A and physical activity		Adjusted Model A and trigger avoidance		Adjusted Model A and acute asthma symptom management		Adjusted Model A and communication	
	OR	95% CI	OR	95% CI	OR	95% CI	OR	95% CI	OR	95% CI	OR	95% CI	OR	95% CI
JDL														
High	Ref.	–	Ref.	–	Ref.	–	Ref.	–	Ref.	–	Ref.	–	Ref.	–
Low	2.05	1.13, 3.72	1.77	.95, 3.33	1.08	.52, 2.22	1.70	.90, 3.20	1.65	.87, 3.13	1.15	.57, 2.34	1.72	.91, 3.26
					− 38.98% change vs. adjusted Model A		− 3.95% change vs. adjusted Model A		− 6.78% change vs. adjusted Model A		− 35.03% change vs. adjusted Model A		− 2.82% change vs. adjusted Model A	
Support														
High	Ref.	–	Ref.	–	Ref.	–	Ref.	–	Ref.	–	Ref.	–	Ref.	–
Low	2.56	1.40, 4.67	2.26	1.19, 4.29	1.90	.93, 3.86	2.19	1.15, 4.18	2.12	1.10, 4.07	2.02	1.02, 4.01	2.09	1.08, 4.03
					− 15.93% change vs. adjusted Model A		− 3.10% change vs. adjusted Model A		− 6.19% change vs. adjusted Model A		− 10.62% change vs. adjusted Model A		− 7.52% change vs. adjusted Model A	
DReAM														
Good	Ref.	–	Ref.	–	Ref.	–	Ref.	–	Ref.	–	Ref.	–	Ref.	–
Adverse	2.33	1.28, 4.23	2.15	1.13, 4.11	1.35	.64, 2.86	1.97	1.02, 3.82	2.00	1.01, 3.96	1.52	.75, 3.07	1.98	1.03, 3.84
					− 37.21% change vs. adjusted Model A		− 8.37% change vs. adjusted Model A		− 6.98% change vs. adjusted Model A		− 29.30% change vs. adjusted Model A		− 7.91% change vs. adjusted Model A	

*OR* odd ratio, *CI* confidence interval, *JDL* job decision latitude, *DReAM* determinants of work-related asthma self-management

^a^Model A: adjusted for age, sex, highest educational degree, body mass index, and smoker status

^b^Model B: adjusted for age, sex, highest educational degree, body mass index, smoker status, physical activity, trigger avoidance, acute asthma symptom management, and communication

Adjusted analyses (Model A) led to an attenuation of all ORs (OR = 1.77; 95% CI 0.95–3.33; OR = 2.26; 95% CI 1.19–4.29; and OR = 2.15; 95% CI 1.13–4.11, respectively). This attenuation was even stronger after additional adjustment for all SMBs (ORs dropped by 39.0%, 15.9%, and 37.2%, respectively), most pronouncedly so when adjusted for acute symptom management (− 35.0%, − 10.6%, and − 29.3%, respectively).

## Discussion

In this study, we found that adverse psychosocial working conditions were associated with poor asthma SMB. Specifically, we observed that low JDL was related to poorer trigger avoidance and acute symptom management, but not to physical activity and communication. Further, low support was related to poorer trigger avoidance, acute symptom management, and communication, but not to physical activity. Finally, the newly created DReAM-scale, which frames working conditions specifically in the context of asthma, related to all types of asthma SMB. Our study also suggests that the above-mentioned working conditions are generally related to poorer asthma control and impaired asthma-specific quality of life. The fact that those associations were attenuated after additional adjustment for asthma SMBs indicates that asthma SMBs may provide an explanatory mechanism.

The results are closely in line with the findings of our qualitative interview-based study [3]. Specifically, a relationship between JDL and communication was neither suggested by our qualitative findings nor found in this study. The same holds true for an association between support and physical activity. All the expected associations could be confirmed with a single exception, that is, a relationship between JDL and physical activity. This contradicts epidemiological studies [25–27] which suggested an association between low JDL and less physical activity. Whereas those studies assessed leisure-time physical activity, the item used in our study included job-related physical activity. Since physical activity was considered as an asthma SMB in terms of symptom prevention by employees with asthma, regardless of whether performed at work or in leisure-time [3], our strategy to assess overall physical activity seemed appropriate. The association between support and different SMBs–especially acute symptom management and communication–was investigated and confirmed before, but not specifically for individuals with asthma [28].

Moreover, psychosocial working conditions were associated with asthma morbidity. This adds to previous results on work stress and asthma morbidity [14], e.g., by using another work stress model than the effort-reward imbalance model applied in that prior study [29]. The participants of our earlier qualitative study did not consider the effort-reward imbalance-components as asthma-relevant working conditions [3].

We present novel insights by documenting that relationships between psychosocial working conditions and asthma morbidity may partly be mediated (and thus explained) by asthma SMB, in particular by acute symptom management and (in case of asthma control) by trigger avoidance.

To our knowledge, this is the first study that statistically confirmed the associations of adverse psychosocial working conditions with poorer asthma-specific SMB at work. To date, prior research has either exclusively relied on qualitative methods [3, 30] or provided statistical estimations which were not specific to employees with asthma [28]. If prospective studies confirm our results and the suspected temporal sequence of the observed associations, it is conceivable to develop interventions to optimize the working conditions for employees with asthma. For instance, patient education programs addressing return-to-work issues could be devised for pulmonary rehabilitation to empower employees with asthma to influence their working conditions. This could be supported by interventions that increase awareness of the importance of JDL and social support among line-managers and employers. Medical staff could support employees with asthma by explaining how to effectively implement asthma SMB at work and raising the awareness that asthma SMBs comprise more than merely trigger avoidance or taking reliever medication. Employees should especially be given the opportunity to manage their acute symptoms, because this seems to be the most problematic domain of asthma SMB (according to our qualitative study [3]) and the most influential on asthma morbidity.

## Limitations

First, this study is cross-sectional and therefore does not provide insights into the temporal nature of the observed associations. Second, since the study focused on chronically ill employees, the results could be affected by the healthy worker effect [31]. The healthy worker effect suggests that individuals with poor health are more likely to drop out of the workforce than apparently healthy employees (for an overview, see Ref. [32]). Third, patients with asthma were included and patients with COPD were excluded based on their admission diagnoses. It remains unclear what criteria were applied to establish the respective diagnoses and if and to what extent COPD was actually ruled out. Since both conditions share some characteristics, they are often confused in the diagnostic process [16]. We originally aimed to check the diagnoses of our participants at discharge from the three cooperating clinics, but this proved unfeasible in the end due to data-security reasons. Therefore, it is possible that some of our participants with asthma do suffer from (comorbid) COPD. Fourth, our overall response rate was low (29.3%) [33], but this was not unusually low in relation to surveys of patients with respiratory conditions [34]. This is partly due to the fact that we did not send out reminders to non-responders or did not ask them repeatedly to complete the questionnaire, because this would have meant unacceptable extra work for the administrational and medical staff in the clinics. In terms of the representativeness of our study sample, it seems reassuring that the gender distribution among our participants (53.8% women) did not differ much from the gender distribution among the overall sample groups in the three clinics (55.6% women). However, the mean age was slightly lower in our sample (50.6 years vs. range of 51.3–55.4 years in the three clinics). Lamentably, we do not have any further information on the overall sample in the three clinics. Therefore, we cannot analyse further possibly influential factors such as additional demographic variables, the profession or job situation. It must be noted that the proportion of participants with uncontrolled asthma according to the Asthma Control Test seemed to be rather high in our sample (65.6%) compared to other studies, which did not specifically focus on rehabilitants (ranging from 44.7 to 59.31%) [14, 21, 35, 36]. Fifth, it is conceivable that physiological conditions at the workplace affect asthma SMB and/or psychosocial working conditions, but we specifically assessed the working conditions which were considered relevant for asthma SMB by the participants of our prior qualitative study [3], e.g., trigger avoidance. Sixth, the objective measurement of SMBs, e.g., of physical activity by using accelerometers, would have delivered less biased data than self-report information. Seventh, although the DReAM-scale, which was specifically developed for this study, showed promising results, it must be noted that some of the items already implied a relationship between working conditions and asthma SMB at work (e.g., “In my daily work routine, I can NOT manage my asthma the way I need to, because there is nobody to take over my tasks”). Thus, the results concerning the relationships between the DReAM-values and the reported asthma SMBs, which were also assessed by self-constructed items, might overestimate the associations.

## Conclusions

Our study found that adverse psychosocial working conditions are associated with poorer asthma SMB at work and increased asthma morbidity. The latter relationship may partly be mediated by asthma SMB. Further research—especially longitudinal studies—is now needed to gain more insights into the complex interrelationships between working conditions, asthma SMB and asthma morbidity and to develop suitable interventions.

## Additional file

**Additional file 1.** Details on measures.

## Abbreviations

CI: confidence interval
COPD: chronic obstructive pulmonary disease
DReAM: determinants of work-related asthma self-management
JDL: job decision latitude
OR: odds ratio
SMB: self-management behaviour
