# Supplementary material for: Psychosocial working conditions, asthma self-management at work and asthma morbidity: a cross-sectional study
Source: Clin Transl Allergy. 2019 May 9;9:25. doi: 10.1186/s13601-019-0264-9 (PMC6509772; doi:10.1186/s13601-019-0264-9)
Supplement: Supplementary file 1 — Additional file 1. Details on measures. [file 13601_2019_264_MOESM1_ESM.docx]

**Additional file 1**

**Measurement of psychosocial working conditions**

JDL and social support at work were measured by the German version of the Copenhagen Psychosocial Questionnaire (1). We combined the scales “Influence at work” (3 items, e.g., “Do you have any influence on what you do at work?”) and “Degree of freedom at work” (2 items, e.g., “Can you decide when to take a break?”) to assess JDL. Those items (Cronbach’s alpha: α=.78) closely resembled the working conditions that affected asthma SMB as identified in our earlier qualitative study (2). Five answer categories were provided which referred to frequencies from “never/hardly ever” to “always”. Scores across items were summed up to a mean score (one missing value accepted) ranging from 0-100 with a higher value indicating a higher JDL. We dichotomized JDL by its tertile (bottom=low JDL, remainder=high JDL). This strategy was applied before in studies dealing with psychosocial working conditions (3-5). The scale “social support” (Cronbach’s alpha: α=.90) comprised four items (e.g., “How often do you get help and support from your colleagues?”) with the same five-tier answer format leading to an equally computed mean (no missing values accepted). Again, those with scores within the bottom tertile were categorized to receive low support while the remainder was categorized as high support.

**Development of the “Determinants of work-related asthma self-management (DReAM)” scale**

We aimed to assess working conditions which patients with asthma perceived as factors influencing their asthma SMB at work in our preceding qualitative study (2). Based on those findings, we generated 36 items (e.g., “My employer is not interested in my asthma”) with a four-tier answer format ranging from “agree completely” to “not at all”.

Next, those items were presented to inpatient rehabilitants at the MEDIAN clinic in Heiligendamm, Germany. A total of 13 employees with asthma who neither participated in the qualitative interview study nor in the questionnaire survey agreed to take part in those cognitive interviews. While they were completing the questionnaire, we asked the participants to share their impression concerning its contents, length and layout and queried a rating of the 36 items (1=very important item to 6=very unimportant item). An additional group of pulmonary inpatients (n=9) provided their rating without being interviewed first. According to the findings from the cognitive interviews, we shortened the questionnaire by deleting some items and rephrased others. Subsequently, all items which received a good rating (1 or 2) by more than 75% of the 22 participants remained in the item pool, whereas all items with a good rating by no more than 50% were deleted. The items in between were evaluated in a theory-guided way. For instance, we aimed to keep at least two items to assess a working condition that was supposed to relate to a given domain of asthma SMB as conceptualised according to Mammen and Rhee (6), e.g., symptom prevention. Based on this approach, the “Determinants of work-related asthma self-management (DReAM)” scale was reduced to 14 items which were included in the questionnaire used for our main data collection (n=221). For statistical analyses, we reversed the coding of four items. Those items assessed adverse working conditions by agreement, whereas the majority of the DReAM scale items measured adverse working conditions by disagreement. We excluded three items with the option “This item does not apply to me” and one item that hardly correlated with the whole scale (=.19). These four items also showed little variation in the sample and reached more than 80% of agreement or did not apply to more than 60% of the sample (10 items remaining). As a next step, we conducted an exploratory factor analysis with orthogonal rotation (Varimax) to identify potential subscales. We deduced the number of potential factors from the scree plot and based on Kaiser’s criterion (7), i.e., considering factors with eigenvalues≥1 only. Further, we eliminated one item with a factor loading<.5 (8) and two items which formed a single factor and were not completely to be differentiated from the primary factor concerning their contents (7 items remaining). The final scale comprised seven items all of which loaded on a single factor (Cronbach’s alpha=.83). For analyses, the DReAM-items (shown in Table 1) were combined into a sum-score ranging from 7-28. Lower values indicated better working conditions that potentially promote asthma SMB at work. We set the cut-off at the top tertile of the score distribution to define adverse working conditions.

**Measurement of asthma self-management**

Since we wanted to analyze different domains of asthma SMB, we grouped the items shown in Table 1 in a theory-guided way (2, 6): one item to assess trigger avoidance, seven items to measure different aspects of acute symptom management, and two items to assess communication. Item scores were added across the subscales acute symptom management and communication, respectively. The two items of the communication domain led to a dichotomization at approx. 80% (approx. 80% with complete fit of needs and conditions (=0) vs. approx. 20% with poor fit of needs and conditions (=1 or 2), no missing values accepted), and we also used this cut-off for the subscale acute symptom management (approx. 80% with good fit of needs and conditions (=0 to 4) vs. approx. 20% poor fit of needs and conditions (=5 to 7), one missing value accepted). We show the items to assess asthma SMB at work in Table 2 together with the numbers and percentages of participants who reported a poor fit of needs and working conditions concerning the different SMBs. The asthma SMBs that led to unfulfilled needs for over 40% of the sample were the following: trigger avoidance at work (60.5%), taking a break at work in case of acute asthma symptoms (52.8%), breathing exercises (46.0%), and leaving an asthma-triggering situation (44.7%).

[Please insert Table 1 near here]

**Measurement of asthma morbidity**

Asthma morbidity was captured by two instruments. First, we used the Asthma Control Test (9) which consists of five items with five response options. The resulting sum-score ranged from 5-25 (one missing value accepted) and was categorized into uncontrolled (<20 points) versus controlled (≥20 points) (10, 11). Second, we measured asthma-specific quality of life using the Marks Asthma Quality of Life Questionnaire (12) which comprised 20 items. The response options ranged from “not at all” to “very severely”. We derived a total score by calculating the mean value across all items ranging from 0-10 with lower values indicating better quality of life (4 missing values accepted), which was dichotomized at its tertile (top tertile=impaired quality of life, remainder=high quality of life) (11).

**References**

1. Nübling M, Stößel U, Hasselhorn H-M, Michaelis M, Hofmann F. Measuring psychological stress and strain at work-Evaluation of the COPSOQ Questionnaire in Germany. GMS Psycho-Social Medicine. 2006;3:Doc05.

2. Heinrichs K, Vu-Eickmann P, Hummel S, Gholami J, Loerbroks A. What are the perceived influences on asthma self-management at the workplace? A qualitative study. BMJ Open. 2018; doi:10.1136/bmjopen-2018-022126.

3. Siegrist J. Effort-reward imbalance at work and health. In: Perrewe P L, Ganster D C, eds. Historical and current perspectives on stress and health, 2002:261–291.

4. Siegrist J, Wahrendorf M. Quality of work, health and early retirement: European comparisons. The individual and the welfare state. Berlin: Springer; 2011. p. 169-177.

5. Loerbroks A, Karrasch S, Lunau T. The longitudinal relationship of work stress with peak expiratory flow: a cohort study. Int Arch Occup Environ Health. 2017;90(7):695-701.

6. Mammen J, Rhee H. Adolescent asthma self-management: a concept analysis and operational definition. Pediat Aller Imm Pul. 2012;25:180-189.

7. Kaiser HF. An index of factorial simplicity. Psychometrika. 1974;39(1):31-6.

8. Cohen J. Statistical power analysis for the behavioral sciences. 2nd. Hillsdale, NJ: Erlbaum; 1988.

9. Nathan RA, Sorkness CA, Kosinski M, et al. Development of the asthma control test: a survey for assessing asthma control. J Allergy Clin Immun. 2004;113:59-65.

10. Schatz M, Sorkness CA, Li JT, et al. Asthma Control Test: reliability, validity, and responsiveness in patients not previously followed by asthma specialists. J Allergy Clin Immun. 2006;117:549-556.

11. Loerbroks A, Leucht V, Keuneke S, Apfelbacher CJ, Sheikh A, Angerer P. Patients’ needs in asthma treatment: development and initial validation of the NEAT questionnaire. J Asthma. 2016;53:427-437.

12. Marks GB, Dunn SM, Woolcock AJ. A scale for the measurement of quality of life in adults with asthma. J Clin Epidemiol. 1992;45:461-472.

**Table 1**: Self-developed items to assess asthma self-management behaviour (n = 221)

| Item | | Poor fit of needs and conditions † | |
| --- | --- | --- | --- |
|  |  | n | % ‡ |
| Trigger avoidance | |  |  |
|  | In my everyday work, I largely avoid the triggers that are relevant to me (e.g., pollen, dust, exercise, smells). (Item does not apply to me: There are no relevant triggers at my workplace.) | 133 | 60.5 |
| Acute asthma symptom management | |  |  |
|  | In case of acute asthma symptoms, I promptly take breaks in my everyday work. | 115 | 52.8 |
|  | In case of acute asthma symptoms, I do breathing exercises in my everyday work. | 99 | 46.0 |
|  | If my asthma gets triggered by a specific situation at work, I withdraw from that situation. | 97 | 44.7 |
|  | In case of acute asthma symptoms, I take up a body posture that eases my breathing in my everyday work. | 67 | 30.9 |
|  | In case of acute asthma symptoms, I deliberately take measures to calm myself in my everyday work. | 62 | 28.6 |
|  | In case of acute asthma symptoms, I get fresh air supply at my workplace as I need to. | 61 | 28.0 |
|  | In case of acute asthma symptoms, I use my emergency inhaler at work. (Item does not apply to me: I do not have an emergency inhaler.) § | 8 | 3.7 |
| Communication | |  |  |
|  | I openly talk to my line-manager about my asthma. | 32 | 14.7 |
|  | I openly talk to my colleagues about my asthma. | 28 | 12.8 |
|  | † = “No, but I would like to” (vs. “Yes, I do this”, “No, I do not need this”, and “This item does not apply to me” = fit of need and working conditions  ‡ of eligible cases  § We re-ran all the analyses that included the acute symptom management sum-score without this item. The odds ratios and confidence intervals did not differ much from the original results. This item was considered as important by the participants of the cognitive interviews and may be relevant among other populations, e.g., individuals who might need their emergency inhaler more regularly, so we decided to report it. | | |
